# Exploring the connection between erythrocyte membrane fatty acid composition and oxidative stress in patients undergoing the Crohn’s disease Therapeutic Diet Intervention (CD-TDI)

**DOI:** 10.1177/17562848251314827

**Published:** 2025-02-16

**Authors:** Natasha Haskey, Clara Letef, James A. Sousa, Munazza Yousuf, Lorian M. Taylor, Derek M. McKay, Christopher Ma, Subrata Ghosh, Deanna L. Gibson, Maitreyi Raman

**Affiliations:** Department of Biology, Irving K. Barber Faculty of Science, University of British Columbia-Okanagan, Kelowna, BC, Canada; Department of Biology, Irving K. Barber Faculty of Science, University of British Columbia-Okanagan, Kelowna, BC, Canada; Gastrointestinal Research Group, Department of Physiology & Pharmacology, Calvin, Phoebe & Joan Snyder Institute for Chronic Diseases, Cumming School of Medicine, University of Calgary, Calgary, AB, Canada; Division of Gastroenterology & Hepatology, Department of Medicine, Cumming School of Medicine, University of Calgary, Calgary AB, Canada; LyfeMD, Calgary, AB, Canada; Gastrointestinal Research Group, Department of Physiology & Pharmacology, Calvin, Phoebe & Joan Snyder Institute for Chronic Diseases, Cumming School of Medicine, University of Calgary, Calgary, AB, Canada; Division of Gastroenterology & Hepatology, Department of Medicine, Cumming School of Medicine, University of Calgary, Calgary, AB, Canada; Department of Community Health Sciences, Cumming School of Medicine, University of Calgary, Calgary, AB, Canada; APC Microbiome Ireland, College of Medicine and Health, University College Cork, National University of Ireland, Cork, Ireland; Department of Biology, Irving K. Barber Faculty of Science, University of British Columbia-Okanagan, Kelowna, BC, Canada; Division of Gastroenterology & Hepatology, Department of Medicine, Cumming School of Medicine, University of Calgary, 6D33 TRW Building, 3280 Hospital Drive NW, Calgary, AB T2N 1N4, Canada

**Keywords:** Crohn’s disease, diet, erythrocyte membrane, fatty acid, oxidative stress

## Abstract

**Background:**

Dietary fatty acids (FA) are crucial to the pathophysiology of inflammatory bowel disease (IBD), influencing systemic and gut inflammatory responses. Dietary FA intake influences the fatty acid profiles of vital cell membranes, which might be a source of inflammatory mediators. Despite their significance, research on dietary FA subtypes and their effects on inflammation and oxidative stress in IBD is limited.

**Objective:**

We investigated the association between dietary FA intake, the erythrocyte membrane FA composition (EMFA), and inflammation and oxidative stress markers in patients with mild–moderate luminal Crohn’s Disease (CD) participating in the CD Therapeutic Dietary Intervention (CD-TDI).

**Design:**

A cross-sectional analysis was performed on 24 participants (13 CD-TDI, 11 habitual diet controls) from a 13-week randomized controlled trial assessing the efficacy of CD-TDI in inducing clinical and biomarker remission in CD.

**Methods:**

EMFA was analyzed using direct-injection gas chromatography, and dietary FA intake was assessed using the ASA 24-h Dietary Assessment Tool^®^.

**Results:**

The CD-TDI group showed a significant increase in dietary n-3 polyunsaturated fatty acids (PUFA) at Week 13 (*p* = 0.04) compared to no changes in the control group. Participants on the CD-TDI also demonstrated a significant reduction in total fat, saturated fat, and arachidonic acid (AA) (*p* < 0.01). EMFA analysis revealed lower percentages of AA (*p* = 0.03) in the CD-TDI group. Positive correlations were observed between C-reactive protein, fecal calprotectin, and dietary stearic acid (*p* < 0.05). Inverse correlations were found between malondialdehyde (MDA) and the Mediterranean Diet Score (*r* = −0.67) as well as MDA and the intake of whole fruit, legumes, and nuts/seeds (*r* > −0.50).

**Conclusion:**

The CD-TDI significantly increased dietary n-3 PUFA intake, reduced pro-inflammatory n-6 PUFA (AA), and improved markers of oxidative stress, supporting its potential in CD management. The cell membrane fatty acid profile might be a therapeutic target in CD.

**Trial registration:**

NCT04596566.

## Introduction

Crohn’s disease (CD) is a chronic relapsing and remitting inflammatory disorder. The primary treatment approaches involve medical therapies, yet up to 46% of patients either do not respond to or lose responsiveness to therapy over time, highlighting a need for alternative treatments.^1^ Emerging evidence highlights the capacity of targeted dietary interventions to shape the gut microbiome, reducing intestinal inflammation and altering disease activity in individuals living with inflammatory bowel disease (IBD).^2^

Fatty acids (FA) play an essential role in health by supporting diverse physiological functions, including maintaining cell membrane integrity, facilitating cell signaling, regulating cellular metabolism, and orchestrating the production of eicosanoids and cytokines.^3^ A growing body of evidence supports that fat type, independent of caloric content, influences intestinal inflammation, metabolism, and host-microbiome function.^4,5^ The primary sources of polyunsaturated fatty acids (PUFAs) in human diets are vegetable and seed oils, rich in linoleic acid (LA), an n-6 PUFA. LA is vital for human tissue composition and is classified as an essential fatty acid; however, when consumed in excess, as observed in the Western diet, it has been associated with various chronic diseases.^6^ Among the n-3 PUFAs, eicosapentaenoic acid (EPA) and docosahexaenoic acid (DHA) are derived from fatty fish, while flax, walnuts, and chia seeds are derived from alpha-linolenic acid.

Dietary FA plays a critical role in IBD pathophysiology, particularly through the modulation of systemic and gut inflammatory responses.^7^ One mechanism involves the metabolism of n-6 PUFAs, which are pro-inflammatory, with LA serving as a precursor to arachidonic acid (AA), thus promoting the generation of oxidized AA metabolites. Moreover, oxidative stress, characterized by an imbalance between pro-oxidants and antioxidants, is intricately linked to inflammatory processes and implicated in the progression and severity of IBD,^8^ with malondialdehyde (MDA) frequently measured as an end-product of lipid peroxidation, especially involving PUFAs like AA.^9^ By contrast, n-3 PUFAs exert their effects by suppressing pro-inflammatory cytokine production.^3^ Notably, the type of saturated fatty acids (SFA) consumed also influences inflammatory responses, with dairy-derived SFAs exhibiting beneficial immunomodulatory effects,^10,11^ while SFAs like palmitic acid derived from palm oil, beef tallow, and meat correlating with the onset of IBD and the exacerbation of inflammation.^12,13^ Despite the importance of dietary intake, fat subtypes, and oxidative stress in IBD, research in this area remains limited.

This work explores the connection between dietary FA intake, erythrocyte membrane FA composition, intestinal and systemic inflammation, as well as oxidative stress (malondialdehyde) in patients with mild to moderately active luminal CD following the Crohn’s Disease Therapeutic Diet Intervention (CD-TDI). To approach this, we quantified the erythrocyte membrane FA composition since it accurately reflects long-term dietary fatty acid intake, given the 120-day lifespan of mature red blood cells (RBCs) in the blood, and compared this to self-reported dietary records.^14^

## Materials and methods

### Crohn’s disease-Therapeutic Diet Intervention

Guided by pilot data and knowledge derived from published literature, the Crohn’s Disease-Therapeutic Diet Intervention (CD-TDI) was formulated.^15^ This comprehensive dietary approach emphasized consuming green leafy vegetables and other foods abundant in beta-carotene, microbial-accessible carbohydrates, and resistant starch.^16^ Rooted in the principles of the Mediterranean diet, the diet is rich in flavanols, targets an optimal n-6:n-3 PUFA ratio (8:1 ratio)^17^ with increased intake of monounsaturated FA (MUFAs) while advised against the consumption of processed foods and food additives.^16^

A cross-sectional analysis was conducted on 24 participants (*n* = 13 CD-TDI, *n* = 11 habitual diet-controls). The analyses were a component of an ongoing 13-week randomized controlled trial designed to assess the efficacy of CD-TDI in inducing clinical and biomarker remission among patients with mild to moderately active luminal CD. The trial employed an intention-to-treat approach to ensure all randomized participants were included in the primary analysis, regardless of adherence. Patients were recruited from outpatient gastroenterology clinics in Calgary and Edmonton, Alberta, Canada, from June 2020 to June 2022. Full details of the study protocol have been previously published.^16^

The trial has been reported in accordance with the CONSORT 2010 guidelines.^18^

### Dietary intake and diet quality indices

Food and beverage intake reported by the participants was assessed at baseline, and week 13 (Wk 13) by two non-consecutive 24-h food recalls using the Automated Self-Administered 24-h (ASA-24^®^) Dietary Assessment Tool (Canadian version).^19^ Details of each food and beverage item, food groups, and nutrient intakes for macro- and micronutrients were downloaded from the ASA-24 researcher website. Guidelines from the US National Cancer Institute were applied for data cleaning. Macronutrients were evaluated in relation to energy intake (per 1000 kilocalories), referred to as adjusted macronutrients.

The Healthy Eating Index (HEI-2020) measures diet quality and adherence to healthy eating patterns^20^ and comprises 13 components: fruits, vegetables, dairy, whole grains, protein foods, fats, and sugars. Higher scores indicate better overall diet quality. The English version of the PREvencion con DIetaMEDiterranea 14-item Mediterranean Diet Adherence Screener was used to calculate a Mediterranean diet score (MDS) from the diet records with one modification.^21^ As alcohol consumption is not routinely advised in IBD, points were not provided for the consumption of alcohol. Using R code,^22^ the HEI score was calculated from the participants’ two non-consecutive 24-h food recalls and Canada’s Food Guide to Healthy Eating^23^ food group serving sizes.

### Blood collection

At baseline and Wk 13, a 6 mL blood sample was collected in a heparinized tube, and an additional 6 mL of blood was collected in a serum separator tube. The heparinized tube was centrifuged at 2000×*g* for 10 min at 4° C for erythrocyte membrane fatty acid composition analyses. One mL of the pellet (i.e., RBCs) was pipetted into a labeled cryotube, placed on ice, and then stored at −80° C until analyses. In the serum separator tube, the blood was allowed to clot before being centrifuged at 12,300×*g* for 15 min. The serum was aliquoted into tubes and stored at −80°C until analysis.

### Long-chain EM phospholipid analysis

Direct-injection gas chromatography was used to quantify long-chain FA from the erythrocyte membrane. FA was extracted using the following combined extraction and methylation protocol. In summary, 500 uL of blood (serum washed off and in heparin solution) was sonicated for 15 s at 40 Hz (Q55 Sonicator, QSonica, Newtown, CT, USA) and then 200 L was transferred to a glass tube and 1.2 mL of hexane and 1.2 mL of 14% boron trifluoride-methanol solution (Sigma-Aldrich - B1127) was added. Samples were heated at 80°C for 90 min. Next, 2 mL of water was added to the samples and centrifuged at 200×*g* for 2 min, and the top hexane layer was removed. The supernatant was injected into a Trace 1300 Gas Chromatograph, equipped with a flame-ionization detector, with AI1310 autosampler (Thermo Scientific, Walkham, MA, USA) in splitless mode. A fused silica Rtx-WAX (Restek, Bellefonte, PA, USA) column 30 m × 0.32 mm coated with 0.5 μm film thickness was used. Helium was supplied as the carrier gas at a flow rate of 1.8 mL/min. The initial oven temperature was 100°C, maintained for 5 min, raised to 240°C at 4°C/min, and then held for 15 min. The flame-ionization detector and the injection port temperature were 280°C and 250°C, respectively. The hydrogen, air, and nitrogen flow rates as makeup gas were 35, 350, and 30 mL/min, respectively. Peak areas and retention times were then calculated using Chromeleon 7 software (Bannockburn, IL, USA) and compared against Supelco 37 Component Fatty Acid Methyl Esters Mix standards (CRM47885, Sigma-Aldrich, MI, USA). Values were expressed as a percent of total fatty acid derived from the area of a single fatty acid peak/total area of peaks.

### Serum malondialdehyde analysis

Serum malondialdehyde (MDA) concentration was analyzed using a commercially available kit (Abcam, ab118970) based on the thiobarbituric acid reactive substances assay and the samples were analyzed according to manufacturer protocol. Briefly, 500 μL of 42 mM H_2_SO_4_ and 125 μL of kit-provided phosphotungstic acid solution were added to 20 μL of serum to precipitate lipids. The solution was incubated at room temperature for 5 min before being centrifuged at 13,000×*g* for 3 min. The pellet was then reconstituted in 200 μL ddH_2_O containing butylated hydroxytoluene. Next, 600 μL of Developer VII/TBA reagent was added to each sample, incubated at 95°C for 60 min, and cooled to room temperature in an ice bath for 10 min. Next, 300 μL of n-butanol was added to each sample to precipitate the MDA-TBA adduct. The sample was vortexed and centrifuged at 16,000×*g* for 3 min, and the top layer was transferred to a new tube. The sample was then dried in a vacuum centrifuge at 40°C, reconstituted in 200 μL ddH2O, and placed in a 96-well plate for analysis. Fluorescence was measured with an excitation wavelength of 532 nm and emission wavelength of 553 nm, and MDA concentration was determined using a standard curve.

### Statistical analysis

Continuous variables were presented as median and interquartile range (IQR), while categorical data were expressed as absolute values and percentages. Paired data were analyzed using the Wilcoxon matched-pairs signed-rank test, and the comparison between groups utilized the Mann–Whitney *U* test. Fisher’s exact test was employed for the comparison of categorical variables. Spearman’s rank-order correlation was conducted to establish correlations between the fatty acid profile of erythrocyte membrane FA and dietary intake and other parameters measured in the study. The statistical package GraphPad Prism Version 9.3.0 (Graph Pad Software, San Diego, CA, USA) was utilized for the analyses and figures.

## Results

### Patient characteristics

Table 1 shows the patient characteristics of the study population. Most participants were overweight with a body mass index of 27 kg/m^2^ (IQR 23–29 kg/m^2^) and 29 kg/m^2^ (IQR 23–33 kg/m^2^) in the CD-TDI and control group, respectively. Participants in the CD-TDI had been diagnosed with CD for a longer duration (median 40 months; IQR 4–156) versus the control (median 8 months, IQR 2–168). Table 2 shows the clinical disease scores and clinical biomarkers of the sample. There were no statistically significant differences between the CD-TDI and the control group at baseline.

**Table 1.** Patient characteristics of the sample (*n* = 24).

Category	CD-TDI baseline (*n* = 13)	Control baseline (*n* = 11)
Demographic information		
Male, *n* (%)	7 (54%)	6 (55%)
Age (years)	37 (31–55)	30 (31–53)
Body mass index (kg/m^2^)	27 (23–29)	29 (23–33)
Disease duration (months)	40 (4–156)	8 (2–168)
Medication		
5-ASA monotherapy, *n* (%)*	5 (38%)	1 (9%)
Biologic, *n* (%)*	3 (23%)	1 (9%)
Corticosteroid, *n* (%)*	1 (8%)	2 (18%)
Immunomodulator, *n* (%)*	3 (23%)	1 (9%)
No therapy, *n* (%)	5 (38%)	5 (45%)

* Used concomitantly with other therapies; data are expressed as medians and quartile 1 and quartile 3 (Med Q1–Q3).

5-ASA, 5-aminosalicylic acid.

**Table 2.** Clinical disease activity and biomarkers of the CD-TDI group versus the control group.

Clinical disease/biomarkers	CD-TDI Baseline (*n* = 13)	CD-TDI Wk 13 (*n* = 13)	Control Baseline (*n* = 11)	Control Wk 13 (*n* = 11)	Between groups *p* Value	
					Baseline^a^	Wk 13^a^
Harvey–Bradshaw Index *p* value within groups	3 (1–5)	1 (0–3)	3 (2–4)	3 (1–4)	0.68	0.16
	0.16^b^		0.75^b^			
C-reactive protein (mg/L) *p* value within groups	1.5 (0.90–3.5)	1.0 (0.70–2.2)	3.0 (1.7–15.0)	4.2 (2.4–15.0)	0.16	**0.002**
	**0.004** ^b^		0.25^b^			
Fecal calprotectin (ug/g) *p* value within groups	137 (34–232) (*n* = 12)	72 (30–153) (*n* = 12)	322 (55–359) (*n* = 10)	258 (120–1770) (*n* = 10)	0.31	**0.03**
	0.10^b^		0.70^b^			

a Mann–Whitney test, *p* < 0.05.

b Wilcoxon matched pairs signed rank test, *p* < 0.05.

The dietary intake of energy (kilocalories (kcal)/day), adjusted macronutrients (grams (g)/1000 kcal), and the total FA are shown in Table 3. In the CD-TDI, there was a significant decrease in energy intake (*p* = 0.05) and a significant increase in adjusted n-3 PUFA intake (*p* = 0.04) from baseline to Wk 13, with no differences in the control. Between groups, there was a significant difference in energy (*p* = 0.03), adjusted carbohydrate (*p* = 0.02), adjusted total fat (*p* = 0.03), and adjusted SFA intake (*p* = 0.002) between the CD-TDI and control at Wk 13. No differences were observed in erythrocyte membrane EMFA composition for total SFAs, MUFAs, and n-6 PUFAs within or between groups at Wk 13. However, n-3 fatty acid and the n-6 to n-3 fatty acid ratios were significantly different at Wk 13 between the CD-TDI and the controls (*p* = 0.005 and *p* = 0.002, respectively).

**Table 3.** Adjusted dietary intake and erythrocyte membrane fatty acid profile of the CD-TDI versus controls.

Variables	CD-TDI^a^ (*n* = 13)			Control^a^ (*n* = 11)			Between Groups^b^ (Wk 13)
Dietary intake (adjusted/1000 kcal)							
	Baseline	Wk 13	*p* Value	Baseline (*n* = 10)	Wk 13	*p* Value	*p* Value
Energy (Kcal)	2112 (1630–2643)	1719 (1376–2220)	**0.05**	2126 (1616–2857)	2508 (2015–3436)	0.70	**0.03**
Protein (g)	35 (33–48)	43 (33–54)	0.54	41 (37–49)	46 (30–53)	>0.99	>0.99
Carbohydrates (g)	116 (106–1357)	134 (122–142)	0.19	117 (99–134)	108 (97–119)	0.56	**0.02**
Total fat (g)	40 (34–51)	35 (29–42)	0.34	38 (32–50)	43 (39–51)	0.38	**0.03**
SFAs (g)	11 (8.9–17)	7 (6.4–11)	0.09	10 (9.7–14)	13 (10–18)	0.28	**0.002**
MUFAs (g)	15 (12–21)	15 (13–17)	0.59	16 (12–18)	17 (15–18)	0.56	0.12
n-6 PUFA (g)	7.9 (6.2–9.6)	8.5 (6.5–9.8)	0.74	8.8 (5.9–14)	9.7 (7.2–11)	0.85	0.40
n-3 PUFA (g)	0.85 (0.71–1.1)	1.4 (0.71–2.3)	**0.04**	0.97 (0.7–1.4)	1.0 (0.95–1.3)	0.23	0.35
n-6:n-3 PUFA ratio	8.4 (6.8–12)	7.6 (4.2–9.2)	0.17	8.9 (6.9–11)	9.0 (7.9–11)	0.51	0.13
Erythrocyte membrane fatty acid profile (% of total fatty acids)							
SFAs	46 (44–49)	47 (43–51)	0.67	46 (45–53)	49 (44–52)	0.63	0.68
MUFAs	17 (16–18)	17 (16–19)	0.39	18 (16–18)	17 (16–19)	0.36	0.78
n-6 PUFA	27 (26–29)	26 (25–31)	0.71	28 (23–30)	26 (25–28)	0.70	0.86
n-3 PUFA	3.7 (2.9–5.0)	4.3 (3.5–5.1)	0.28	3.2 (2.3–4.0	3.1 (2.5–3.7)	0.70	**0.005**
n-6:*n*-3 PUFA ratio	7.0 (4.2–8.5)	5.5 (4.8–6.4)	0.17	7.6 (6.9–9.2	7.3 (6.2–9.3)	0.51	**0.002**

a Wilcoxon matched signed rank test, *p* < 0.05.

b Mann–Whitney U test, *p* < 0.05.

Data are expressed as medians and quartile 1 and quartile 3 (Med Q1–Q3). Not normally distributed variables.

kcal, kilocalories; MUFAs, monounsaturated fatty acids; PUFAs, polyunsaturated fatty acids; SFAs, saturated fatty acids; Wk 13, week 13.

Table 4 shows the FA subtypes for adjusted dietary intake and the erythrocyte membrane. Significantly reduced dietary intake of the SFAs, myristic acid (*p* = 0.005), palmitic acid (*p* = 0.002), stearic acid (*p* = 0.007) and the n-6 PUFA, AA (*p* = 0.0001) and DHA (*p* = 0.05) were observed between the CD-TDI and controls at Wk 13. From baseline to Wk 13, the CD-TDI had a significantly lower intake of myristic acid (*p* = 0.05). In the erythrocyte membrane of the CD-TDI group, within-group differences at Wk 13 showed a lower percentage of AA (n-6 PUFA; median 0.09% vs 0.07%, *p* = 0.03), and a significant increase in oleic acid (MUFA; median 11% vs 12%, *p* = 0.02). Between-group differences at Wk 13 showed a significantly lower level of palmitoleic acid (median 0.18% vs 0.25% *p* = 0.04) and a significantly higher intake of DHA (n-3 PUFA; median 3.7% vs 2.6%, *p* = 0.02) in the CD-TDI group versus the control group.

**Table 4.** Dietary intake and erythrocyte membrane fatty acid profile of the fatty acid sub-types for CD-TDI versus controls.

	CD-TDI^a^ (*n* = 13) Median (Q1–Q3)			Control^a^ (*n* = 11) Median (Q1–Q3)			Between Groups^b^ (Wk 13)
Dietary intake of fatty acids (adjusted/1000 kcal)							
Fatty acids	Baseline	Wk 13	*p* Value	Baseline (*n* = 10)	Wk 13	*p* Value	*p* Value
14:0 Myristic acid	0.89 (0.46–1.8)	0.56 (0.20–0.64)	**0.05**	0.81 (0.32–1.6)	1.3 (0.58–1.8)	0.23	**0.006**
16:0 Palmitic acid	6.3 (5.3–8.2)	4.5 (4.0–6.3)	0.13	6.1 5.6–7.4)	6.9 (6.2–9.2)	0.23	**0.002**
16:1 Palmitoleic acid	0.52 (0.42–0.64)	0.49 (0.35–0.61)	0.38	0.62 (0.45–0.66)	0.71 (0.35–0.88)	0.49	0.09
18:0 Stearic acid	2.6 (2.0–3.6)	1.5 (1.3–2.7)	0.15	2.6 (2.0–3.4)	2.8 (2.5–4.0)	0.32	**0.007**
18:1n9 Oleic acid	14 (11–20)	14 (11–16)	0.59	15 (11–17)	16 (14–16)	0.77	0.19
18:2 Linoleic acid	6.3 (5.4–8.4)	6.2 (5.3–8.0)	0.74	7.5 (4.9–12)	8.0 (6.2–9.3)	0.70	0.09
18:3n3 α-Linolenic acid	0.81 (0.59–1.0)	1.1 (0.62–1.9)	0.27	0.93 (0.50–1.1)	1.0 (0.83–1.2)	0.07	0.95
20:4n-6 Arachidonic acid	0.07 (0.04–0.12)	0.09 (0.06–0.17)	0.13	0.07 (0.04–0.11)	0.10 (0.04–0.10)	0.92	**<0.0001**
20:5n-3 Eicosapentaenoic acid	0.003 (0.001–0.07)	0.006 (0.002–0.08)	0.22	0.005 (0.001–0.02)	0.005 (0.001–0.02)	0.92	0.61
22:2n-3 Docosahexaenoic acid	0.02 (0.006–0.04)	0.08 (0.03–0.15)	0.11	0.023 (0.01–0.06)	0.029 (0.007–0.06)	>0.99	**0.05**
Erythrocyte membrane fatty acid profile (% of total fatty acids)							
14:0 Myristic acid	1.5 (1.1–2.4)	1.7 (1.2–1.9)	>0.99	1.4 (1.3–1.4)	1.2 (1.1–2.3)	0.97	0.45
16:0 Palmitic acid	26 (26–28)	28 (15–29)	0.17	28 (26–29)	27 (26–29)	0.58	0.77
16:1 Palmitoleic acid	0.17 (0.13–0.35)	0.18 (0.12–0.25)	0.38	0.24 (0.17–0.36)	0.25 (0.19–0.23)	0.78	**0.04**
18:0 Stearic acid	16 (14–18)	15 (14–19)	0.67	16 (14–20)	17 (13–20)	0.55	0.61
18:1n-9 Oleic acid	11 (10–13)	12 (11–15)	**0.02**	11 (10–14)	12 (11–12)	0.49	0.39
18:2n-6 Linoleic acid	8.3 (7.5–10)	8.4 (7.5–10)	0.67	8.9 (7.2–9.5)	8.8 (7.9–9.8)	0.76	0.73
18:3n-6 γ-Linolenic acid	0.08 (0.04–0.17)	0.06 (0.03–0.09)	0.18	0.05 (0.04–0.09)	0.05 (0.04–0.14)	0.89	0.63
18:3n-3 α-Linolenic acid	0.07 (0.02–0.79)	0.15 (0.09–0.29)	0.61	0.10 (0.05–0.22)	0.09 (0.06–0.24)	0.78	0.42
20:0 Arachidic acid	0.49 (0.34–0.83)	0.78 (0.38–1.4)	0.34	0.72 (0.42–1.2)	0.93 (0.33–1.3)	0.90	0.79
20:3n-9 Eicostrienoic acid	1.0 (0.72–1.2)	0.92 (0.74–1.1)	0.79	1.1 (0.81–1.3)	1.1 (0.80–1.3)	0.78	0.25
20:4n-6 Arachidonic acid	0.09 (0.07–0.52)	0.07 (0.05–0.14)	**0.03**	0.08 (0.04–0.14)	0.06 (0.02–0.11)	0.54	0.48
22:4n-6 Dihomo-γ-linolenic acid	13 (12–14)	12 (12–14)	0.80	14 (13–15)	13 (13–14)	0.95	0.73
20:5n-3 Eicosapentaenoic acid	0.45 (0.25–0.85)	0.45 (0.32–1.1)	0.45	0.37 (0.11–0.80)	0.26 (0.20–0.72)	0.43	0.23
22:2n-3 Docosahexaenoic acid	2.8 (2.1–3.2)	3.7 (2.8–4.3)	0.08	2.7 (2.1–3.0)	2.6 (2.1–2.9)	0.81	**0.02**
Oxidative stress							
Malondialdehyde (nmol/ml)	9.3 (7.9–10)	9.3 (7.9–11)	0.90	9.3 (8.4–11)	11 (8.1–14)	0.23	0.17

Data are expressed as medians and quartile 1 and quartile 3 (Med Q1-Q3). Not normally distributed variables.

a Wilcoxon matched signed rank test, *p* < 0.05.

b Mann–Whitney *U* test, *p* < 0.05.

kcal, kilocalories; Wk 13, week 13.

**Figure 1.**
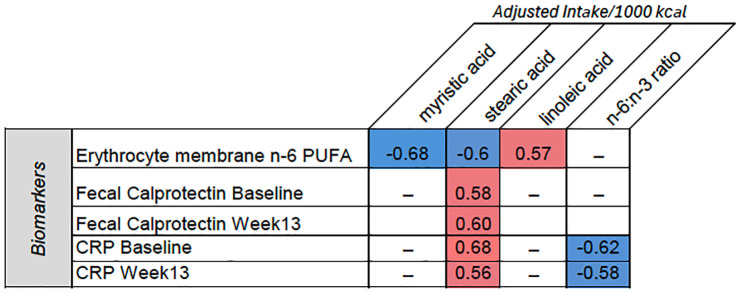
Significant correlations between biomarkers and adjusted dietary intake of fatty acids (per 1000 kcal). The figure shows correlations for erythrocyte membrane n-6 PUFA, fecal calprotectin at baseline and week 13, and CRP at baseline and week 13 with myristic acid, stearic acid, linoleic acid, and the n-6:n-3 ratio. Blue cells represent negative correlations, while red cells represent positive correlations. Spearman-rank correlation, *p* < 0.05. CRP, C-reactive protein; n-6 polyunsaturated fatty acids.

Table 5 shows the HEI scores within and between groups. A higher HEI score indicates the diet aligns with national diet recommendations. Despite significant differences in the moderation HEI and saturated fat HEI between groups, there was a lack of association between HEI scores and erythrocyte membrane fatty acid composition. Apart from a positive association between the erythrocyte membrane MUFA and the moderation HEI (*r* = 0.59, *p* = 0.04, baseline), no other differences were noted. A Spearman’s rank-order correlation was completed to explore possible relationships between dietary intake components and erythrocyte membrane fatty acid composition (Figure 1). In the CD-TDI group at Wk 13, the erythrocyte membrane n-6 PUFA negatively correlated with dietary intake of the SFAs, myristic acid (*p* = 0.02), and trend for stearic acid (*p* = 0.06), while positively correlated with dietary LA (*p* = 0.05). These observations were not observed in the control group (*p* > 0.05). Markers of inflammation, C-reactive protein (CRP), and fecal calprotectin were positively associated with dietary stearic acid consumption (*p* < 0.05) but not erythrocyte membrane FA composition. No other correlations were observed between diet, the erythrocyte membrane, or inflammatory biomarkers (*p* > 0.05). Unexpectedly, the dietary n6:n3 PUFA ratio at Wk 13 (median 5.5:1) in the CD-TDI was negatively associated with the CRP at baseline and Wk 13 (*p* = 0.03 and *p* = 0.04, respectively).

**Table 5.** Healthy Eating Index Scores of CD-TDI and controls at baseline and week 13.

Variables	CD-TDI^a^ (*n* = 13) Median (Q1-Q3)			Control^a^ (*n* = 11) Median (Q1-Q3)			Between Groups^b^ (Wk 13)
	Baseline	Wk 13	*p* Value	Baseline (*n* = 10)	Wk 13	*p* Value	*p* Value
Total HEI	70 (65–79)	77 (69–82)	0.15	68 (65–77)	63 (61–78)	**0.05**	0.07
Moderation HEI	27 (22–31)	31 (29–36)	**0.05**	29 (23–29)	24 (18–28)	0.73	**0.001**
Saturated Fat HEI	8 (2.0–9.5)	10 (8–10)	0.13	8.5 (3.3–9.0)	6.0 (0–9)	0.45	**0.007**
Unsaturated HEI	10 (10–10)	10 (9.9–10)	0.63	10 (10–10)	10 (10–10)	>0.99	0.60

HEI, healthy eating index; Wk 13, week 13.

Data are expressed as medians and quartile 1 and quartile 3 (Med Q1–Q3). Not normally distributed variables. ^a^Wilcoxon matched signed rank test, *p* < 0.05. ^b^Mann–Whitney *U* test, *p* < 0.05.

After noting a reduction in AA in the erythrocyte membrane of the CD-TDI group, alongside notable distinctions in dietary intake of AA between the CD-TDI and control group, we opted to explore correlations between oxidative stress (malondialdehyde, MDA) and inflammatory markers (CRP/fecal calprotectin). MDA was not significantly different between the CD-TDI and control groups at baseline (CD-TDI: 9.3; IQR 7.9–10 and control: 9.3; IQR 8.4–11) and Wk 13 (CD-TDI: 9.3; IQR 7.9–11 and control: 11; IQR 8.1–13; *p* >0.05; Table 4). While not reaching statistical significance, it is noteworthy that MDA levels showed an observable increase of 8% in the control group, whereas no such increase was observed in the CD-TDI.

We further analyzed MDA to ascertain its correlation with dietary intake in the CD-TDI group (Table 6). Our findings revealed a positive correlation between serum MDA levels and the n6:n3 PUFA ratio, both in terms of baseline erythrocyte membrane FA composition (*r* = 0.65) and dietary intake (*r* = 0.55). Conversely, we observed an inverse correlation with the MDS (*r* = −0.67), suggesting that a diet of higher quality could mitigate the increase in MDA levels. Notably, specific food groups, including whole fruits, legumes, nuts and seeds, and meat alternatives, exhibited inverse associations with the elevation of MDA levels. A similar analysis was completed in the control group; however, except for a positive correlation in MDA and fruit juice intake (*r* = 0.59), all other correlations were very weak (*r*-value between 0 and 0.36 or 0 and −0.36).

**Table 6.** Spearman rank-order correlation for CD-TDI for malondialdehyde and diet.

Variables	Malondialdehyde	
	*p* Value	Spearman *R*
*Fatty Acid Ratio_baseline*:		
Erythrocyte membrane n-6:n-3 PUFA ratio	0.02	0.65
Dietary n-6:n-3 PUFA ratio	0.06	0.55
*Healthy Eating Index (HEI)_Wk 13*:		
Unsaturated HEI^a^	0.02	−0.62
*Mediterranean Diet Score_Wk 13*:		
Total score^b^	0.02	−0.67
Legumes	0.01	−0.76
*Food Groups_Wk 13*:		
Whole Fruit^c^	0.06	−0.53
Legumes (vegetables)^d^	0.01	−0.73
Meal alternates^e^	0.02	−0.63
Nuts and seeds^f^	0.03	−0.60
Legumes (protein)^g^	0.03	−0.73

Spearman-rank correlation, *p* < 0.05.

a Based on the intake of healthy unsaturated fats, such as those found in nuts, seeds, avocados, and vegetable oil.^21^

b Based on the PREvencion con DIetaMEDiterranea 14-item Mediterranean Diet Adherence Screener.^18^

c Intact fruits (whole or cut), excluding citrus, melons, and berries.

d Beans and peas (legumes) computed as vegetables (cup eq.).

e Canadian Food Group meat and alternative servings, which include red meat, fish, shellfish, poultry, eggs, legumes, and nut butters.^20^

f Peanuts, tree nuts, and seeds; excludes coconut (oz. eq.).

g Beans and peas (legumes) computed as protein foods (oz. eq.).

EM, erythrocyte membrane.

## Discussion

To the best of our knowledge, this is one of the first studies to examine erythrocyte membrane FA and their association with dietary fatty acid intake, diet quality, and inflammatory markers (CRP and fecal calprotectin) and oxidative stress (MDA) in patients with CD undergoing a dietary intervention.

Our findings reveal a significant decrease in dietary SFAs, including myristic, palmitic, and stearic acids, in the CD-TDI group compared to the control group. This reduction in SFA intake is attributed to the CD-TDI’s exclusion of beef, dairy products, and fats such as palm oil, beef tallow, cocoa butter, and lard, which are rich sources of these SFAs. Despite the alterations in dietary habits, no discernible shifts were noted in the levels of SFAs within the erythrocyte membrane. Others have also observed similar findings, noting significant variations in dietary intake of SFAs and their corresponding levels in the erythrocyte membrane.^24^

In contrast to the SFAs, the PUFA content of erythrocyte membranes reflects n-6 PUFA dietary intake.^24,25^ Our observations revealed a significant reduction in the n-6 PUFA and AA in the erythrocyte membrane of the CD-TDI group, with significant differences in dietary intake of AA between the CD-TDI and control groups. The CD-TDI emphasizes the avoidance of vegetable oils (safflower, sunflower, and soybean oils), red meat, dairy products, and processed foods which are rich sources of LA. Several studies demonstrate that individuals who consume foods rich in AA, such as red meat, vegetable oils derived from soya, sunflower, canola, and corn, as well as margarine, have higher tissue levels of AA.^26–28^ Higher tissue levels of AA have been implicated in the pathophysiology of IBD and are connected to higher levels of inflammation.^29,30^ To balance the fatty acid profile in the CD-TDI, increased intake of MUFA (e.g., olive oil) and n-3 PUFA were encouraged. While dietary MUFA intake did not change, the incorporation of oleic acid (MUFA) within the erythrocyte membrane was observed. We speculate that the inconsistency between reported dietary oleic acid intake and the composition of erythrocyte membranes could be attributed to underreporting. Given that olive oil, a significant source of oleic acid is often added to foods, it might have been inadvertently omitted from dietary recalls. In terms of the erythrocyte membrane and the consumption of n-3 PUFAs in the diet, we observed that the CD-TDI exhibited higher levels of DHA compared to the control diet. The CD-TDI emphasizes the consumption of n-3 PUFA-rich foods like fatty fish, flaxseeds, and chia seeds while maintaining a reduced intake of n-6 PUFAs. In summary, identifying MUFA and n-3 PUFA levels in erythrocyte membranes serves as an objective biomarker reflecting adherence to the CD-TDI.

Studies in IBD indicate an increased concentration of dietary n-3 PUFA is associated with reduced inflammation and improved disease activity.^12,31^ While no significant association was observed in our cohort between dietary or erythrocyte membrane n-3 PUFAs with CRP or fecal calprotectin, a notable limitation in our study is the patient population with mild systemic inflammation (CRP <5 mg/L). Further investigation into the impact of n-3 PUFAs on inflammation and disease activity is warranted, particularly in patients with increased disease severity. In addition, exploring the effects of n-3 PUFAs on various pro-inflammatory cytokines such as TNF-α, IL-1α, IL-6, IL-8, and IFN-γ would provide valuable insights.

Malondialdehyde (MDA) serves as a biomarker to measure oxidative stress among various chronic diseases, including IBD.^9^ An intriguing finding in our CD-TDI group was the observed inverse relationship between adherence to the Mediterranean diet and serum MDA levels. This relationship was further evident in specific components of the Mediterranean diet, including nuts, seeds, whole fruit, and legumes, suggesting their potential protective effect against oxidative stress. Notably, these components, such as whole fruit (such as apples and bananas), nuts and seeds (like flaxseed, chia seeds, and almond butter), and legumes (such as lentils), are integral parts of the CD-TDI, emphasizing their importance in promoting health and reducing oxidative stress. We are not aware of any previous studies in IBD showing an influence of the Mediterranean diet or food components and its link to MDA levels. However, studies in healthy individuals have shown that high consumption of fruit and vegetables and adherence to the Mediterranean diet are inversely associated with MDA levels.^32,33^ Further work is needed in this area, with the potential to explore additional oxidative stress markers, such as total antioxidant status, glutathione (GSH), 8-hydroxy-2′–deoxyguanosine, and clinical treatment response in dietary intervention trials.^8,34^

The “Western diet” is highly associated with the pathogenesis of many inflammation-mediated modern chronic diseases, including IBD.^35^ The intake of n-6 PUFAs exceeds the volume of n-3PUFAs, and as a result, the n-6/n-3 ratio has substantially risen from 1:1 in our ancestral diets to around 20–50:1 in today’s Western diets.^35^ This change in the n-6/n-3 ratio, possibly more than any other dietary factor, has contributed to the significant increase in the prevalence of body tissue and systemic inflammation.^35^ Our previous work demonstrated that the optimal ratio of n-6:n-3 PUFA is 8:1 in patients with IBD and was associated with reduced gut and systemic inflammation.^17^ The CD-TDI decreased n-6:n-3 PUFA ratio in the erythrocyte membrane decreased from 7:1 (1QR: 6.8–12) to 5:5 (IQR: 4.2–9.2); however, the dietary n-6:n-3 PUFA ratio did not differ. This discrepancy underscores the potential inaccuracies inherent in self-reported food recalls. We observed no association between fecal calprotectin and the n-6:n-3 PUFA ratio; however, unexpectedly, we found an inverse correlation between the n6:n3 PUFA ratio and CRP. While the n-6:n-3 PUFA ratio can serve as a useful guideline for assessing dietary balance, it is crucial to consider the broader context. The focus on the ratio may oversimplify the complex biological activities of individual n-6 and n-3 FA, as each type of fatty acid has unique functions and influences various physiological processes. For example, variations in genetic makeup, such as in the genetic variants in the fatty acid desaturase (FADS) genes, along with factors like age, gender, and health status, can influence the optimal n-6:n-3 PUFA ratio in one’s diet. A comprehensive understanding of the specific FA, their sources, and their diverse roles in the body is essential for a more accurate interpretation of the n-6:n-3 PUFA ratio.^36^ Ultimately, tailored dietary recommendations, both personalized and population-based, may be necessary to promote optimal nutrition and well-being.

The HEI evaluates diet quality and the quality of various dietary components, including fatty acids.^37^ Despite modifications in dietary HEI scores, we found no discernible correlation between HEI and the erythrocyte membrane FA composition. This incongruity may stem from limitations within the HEI scoring system. For instance, while the HEI acknowledges the importance of certain fatty acid ratios (such as the ratio of PUFA and MUFA to SFAs),^37^ it fails to differentiate between specific types of FA, such as those derived from omega-3 PUFA, known for their significant health benefits. Notably, similar discrepancies have been noted by other researchers in existing literature.^38^

Our study has several limitations. First, the cross-sectional design prevents us from establishing causal relationships. Another limitation is the patient population with mild systemic inflammation (CRP <5 mg/L). Consequently, this allowed us to examine individuals with mild disease, which limits the generalizability of our findings to populations with more severe disease or different inflammatory profiles. Finally, we acknowledge that this limited temporal analysis provides only a snapshot of the association at two time points and suggest that future studies with more frequent measurements could strengthen the interpretation of these correlations over time. To advance our understanding of the intricate relationship between fat subtypes and inflammatory processes in IBD, it is imperative to address critical research gaps. First, the development of improved dietary assessment techniques that precisely quantify FA types and validate these measurements against biomarker data is urgently needed. Furthermore, exploring novel biomarkers and analytical techniques is imperative for assessing serum and erythrocyte membrane levels of fatty acid subtypes to understand their dynamic changes over time and their correlation with clinical endpoints. Lastly, mechanistic studies are needed to unravel the relationships between FA subtypes and inflammatory markers and the impact of dietary interventions on inflammation modulation. By addressing these research gaps, we can advance our understanding of the complex interplay of FA and inflammatory processes, paving the way for more targeted and effective nutritional interventions to enhance the health of patients with IBD.

## Conclusion

In conclusion, this study highlights the impact of CD-TDI on fatty acid intake and its association with inflammatory markers and oxidative stress in patients. Adherence to the diet resulted in a significant increase in n-3 PUFAs, particularly DHA while reducing the intake of pro-inflammatory AA, an n-6 PUFA. This change in dietary intake was reflected in the erythrocyte membrane composition. The study also identified an inverse relationship between the Mediterranean diet components and the oxidative stress marker—MDA, suggesting the potential protective effect of specific foods like fruits, nuts, seeds, and legumes.

## Supplemental Material

sj-doc-2-tag-10.1177_17562848251314827 – Supplemental material for Exploring the connection between erythrocyte membrane fatty acid composition and oxidative stress in patients undergoing the Crohn’s disease Therapeutic Diet Intervention (CD-TDI) Supplemental material, sj-doc-2-tag-10.1177_17562848251314827 for Exploring the connection between erythrocyte membrane fatty acid composition and oxidative stress in patients undergoing the Crohn’s disease Therapeutic Diet Intervention (CD-TDI) by Natasha Haskey, Clara Letef, James A. Sousa, Munazza Yousuf, Lorian M. Taylor, Derek M. McKay, Christopher Ma, Subrata Ghosh, Deanna L. Gibson and Maitreyi Raman in Therapeutic Advances in Gastroenterology

sj-docx-1-tag-10.1177_17562848251314827 – Supplemental material for Exploring the connection between erythrocyte membrane fatty acid composition and oxidative stress in patients undergoing the Crohn’s disease Therapeutic Diet Intervention (CD-TDI) Supplemental material, sj-docx-1-tag-10.1177_17562848251314827 for Exploring the connection between erythrocyte membrane fatty acid composition and oxidative stress in patients undergoing the Crohn’s disease Therapeutic Diet Intervention (CD-TDI) by Natasha Haskey, Clara Letef, James A. Sousa, Munazza Yousuf, Lorian M. Taylor, Derek M. McKay, Christopher Ma, Subrata Ghosh, Deanna L. Gibson and Maitreyi Raman in Therapeutic Advances in Gastroenterology

## Appendix

### Abbreviations

AA Arachidonic acid

CD Crohn’s disease

CD-TDI Crohn’s disease therapeutic diet intervention

CRP C-reactive protein

DHA Docosahexaenoic acid

EPA Eicosapentaenoic acid

FA Fatty acids

FCP Fecal calprotectin

HEI Healthy Eating Index

IBD Inflammatory bowel disease

LA Linoleic acid

MDA Malondialdehyde

MDS Mediterranean Diet Serving

MUFA Monounsaturated fatty acid

PUFA Polyunsaturated fatty acid

n-6 PUFA Omega-6 polyunsaturated fatty acid

n-3 PUFA Omega-3 polyunsaturated fatty acid

RBC Red blood cell

ROS Reactive oxygen species

SFA Saturated fatty acid

Wk 13 Week 13

## Declarations

**Ethics approval and consent to participate:** The research protocol was approved by the Conjoint Health Research Ethics Board at the University of Calgary (REB19-0402) and the Health Research Ethics Board—Biomedical Panel at the University of Alberta (Pro00090772). Written informed consent was obtained from all subjects involved in the study.

**Consent for publication:** The participating patients gave consent to publish the analyzed data in an anonymized form.

**Availability of data and materials:** The data supporting this study’s findings are available upon request. Please contact the corresponding author for further details.
