# Supplementary material for: Exploring the connection between erythrocyte membrane fatty acid composition and oxidative stress in patients undergoing the Crohn’s disease Therapeutic Diet Intervention (CD-TDI)
Source: Ther Adv Gastroenterol. 2025 Feb 16;18:17562848251314827. doi: 10.1177/17562848251314827 (PMC11831646; doi:10.1177/17562848251314827)
Supplement: sj-doc-2-tag-10.1177_17562848251314827 – Supplemental material for Exploring the connection between erythrocyte membrane fatty acid composition and oxidative stress in patients undergoing the Crohn’s disease Therapeutic Diet Intervention (CD-TDI) [file sj-doc-2-tag-10.1177_17562848251314827.doc]

**
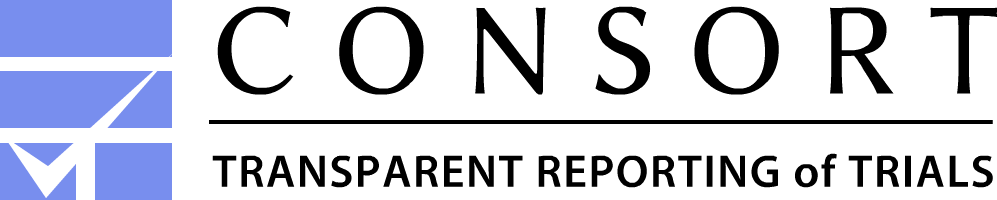
**

**CONSORT 2010 Flow Diagram for UC Study**

**Allocation**

**Analysis**

**Follow-Up**

**Enrollment**

Assessed for eligibility (n=106)

Excluded (n=78)

♦  Not meeting inclusion criteria (n= 5)

♦  Declined to participate (n=49)

♦  No response (n=14)

♦  Dropped prior to randomization (n=10)

Analysed (n=13)
♦ Excluded from analysis (n=0)

Discontinued intervention (give reasons) (n=3)

Allocated to intervention (n=16)

♦ Received allocated intervention (n=16)

Discontinued Study (give reasons) (n=1)

Allocated to control (n=12)

♦ Received normal standard of care (n=12)

Analysed (n=11)
♦ Excluded from analysis (n=0)

Randomized (n=28)
